# Heterogeneous Catalytic Oxidation of Amides to Imides by Manganese Oxides

**DOI:** 10.1038/s41598-018-31729-3

**Published:** 2018-09-11

**Authors:** Sourav Biswas, Harshul S. Khanna, Quddus A. Nizami, Donald R. Caldwell, Katherine T. Cavanaugh, Amy R. Howell, Sumathy Raman, Steven L. Suib, Partha Nandi

**Affiliations:** 1 Department of Chemistry, University of Connecticut, Storrs, CT 06269 USA; 2 Institute of Materials Science, University of Connecticut, Storrs, CT 06269 USA; 3 Corporate Strategic Research, ExxonMobil Research and Engineering Company, 1545 US 22 East, Annandale, NJ 08801 USA; 4 Present Address: Department of Chemistry, University of Wisconsin-Madison, 1101 University Avenue, Madison, WI 53706 USA

## Abstract

Herein, we report a one-step peroxide mediated heterogeneous catalytic oxidation of amides to imides utilizing a series of manganese oxides. Among them, Cs/Mn_2_O_3_ was found to be the most active catalyst for the selective partial oxidation of *N*-benzylbenzamide to diphenyl imide. We have been able to apply an optimized oxidation method to other aromatic substrates. The feasibility of using air as an oxidant, the heterogeneous nature, inexpensive catalytic materials, respectable turnover numbers, and chemoselectivity to imides make this methodology an attractive choice for functional group transformations of amides to imides.

## Introduction

Imide functional groups have versatile applications in pharmaceutical, polymer, and natural product synthesis^1,2^. Traditionally imides have been synthesized by reaction between dicarboxylic acid e.g., phthalic acid (or anhydride) and amines^3^. Acylation of amides^4^, and rearrangement of isocyanates can also yield imides^5^. A few homogeneous catalytic oxidations of amides to imides are reported recently using organic hydroperoxides as terminal oxidants^6–8^.

Manganese oxides have gained wide recognition as powerful materials in catalytic oxidation reactions^9,10^. Readily accessible multiple oxidation states, high abundance, thermally stable structural forms, and dioxygen reduction ability are some of the important properties of these materials^11–13^. Several promoter ions can reside as structural or charge balancing ions to enhance the catalytic activity of manganese oxides^14–16^. Moreover, the diversity of synthetic methods allows preparation of different forms of manganese oxide of varying porosity and crystallinity such as octahedral molecular sieves (OMS), octahedral layer (OL), amorphous manganese oxide (AMO), birnessite, α-manganese oxide, and mesoporous manganese oxide^11^. These mixed valent, high surface area materials have excellent redox properties which lead to highly efficient manganese based oxidation catalysts. The effect of ion promotion in the manganese oxides has been studied previously^17^. One proposed mechanism is that with cesium ion promotion in manganese oxides, the surface tends to induce a more basic character, which allows the binding energy for lattice oxygen to be lowered^17,18^. Manganese oxide based materials have been utilized in different types of catalytic oxidation reactions such as selective oxidation of alcohols to aldehydes^19^, hydrocarbons to alcohols and ketones^20^, styrenes to styrene oxides^21^, alcohols to amides^22–26^, and amines to imines^27–29^. Furthermore, manganese oxide catalysts are effective in oxidative coupling of alkynes^30^, alkyne-silanes^31^, and alkyne-amine^32^. Herein, we report a heterogeneous catalytic oxidation of benzylic amides to imides by two different classes of manganese oxides.

## Results and Discussion

### *N*-benzylbenzamide Oxidation

Initially we utilized the oxidation of *N*-benzylbenzamide as the model reaction for developing the optimal reaction conditions^33^. In our early screening attempts, we used a number of redox active metal oxides (e.g., BiVO_4_, CuMoO_4_, CoMoO_4_) without much success. In a few cases, over-oxidation of the imide and thermal cracking reactions were observed. This was, however, not the case with Mn oxides. Building on our recently employed mesoporous manganese oxide material as a highly active oxidation catalyst^17,34–36^, a minute amount (0.14%) of electropositive Cs ions has been introduced in the manganese oxide structure, which enhanced the oxidation ability of manganese oxide by several orders of magnitude (100 fold) in oxidations of alcohols to aldehydes and amines to imines^17,35^. Herein, Cs^+^ promoted mesoporous manganese oxide (meso Cs/Mn_2_O_3_) was used as a model catalyst and acetonitrile as the model solvent. In early attempts, the use of air and oxygen as the oxidant did not produce any product (Entries 1 and 2, Table 1). Using H_2_O_2_ also did not give any product (Entry 3, Table 1). We then switched our attention to *tert*-butyl hydroperoxide (TBHP in water) as the oxidant. TBHP is a popular oxidant for oxidation of inert C-H bonds due to high thermal stability and solubility^37,38^ as compared to H_2_O_2_. The reaction was performed then with different loadings of catalyst and TBHP (Entries 1–4, Table S1). The reaction was then performed with solvents of different polarities at their respective boiling points (Entries 4–8, Table S1). No imide was detected using dioxane, THF, or toluene. Chloroform and CH_2_Cl_2_ were avoided due to potential generation of COCl_2_. (Entry 8, Table S1). Consequently, acetonitrile (ACN) was chosen as solvent for further reactions. The formation of another product (benzamide) can be attributed to the hydrolysis of intermediates.

**Table 1:** Optimization of oxidation of *N*-benzylbenzamide.

Entry	Oxidant	Additives	Conv. (%)^b^	Selectivity (%)^b^	
				Diphenyl Imide	N-benzamide
1	Air	none	0	nd	nd
2	Oxygen	none	0	nd	nd
3	H_2_O_2_	none	0	nd	nd
4	TBHP/water	none	5	50	22
5	TBHP/nonane	none	10	15	80
6^c^	TBHP/nonane	NHPI^d^	25	90	10
7^e^	TBHP/nonane	NHPI	90 (85)	95	5
8	THBP/nonane	Air	10	15	80
9	TBHP/nonane	Argon	0	nd	nd
10^e^	None	NHPI	5	80	10
11	None	NHPI	0	nd	nd
12^f^	TBHP/nonane	NHPI	0	nd	nd

^a^Reaction conditions: *N*-benzyl benzamide (100 mg, 0.5 mmol, 1.0 equiv), meso Cs/Mn_2_O_3_ (50 mg), acetonitrile (5 mL, 95 mmol, 190 equiv), 80 °C, TBHP (5 mmol, 0.45 g, 10 equiv), 22 h. ^b^Conversions and selectivities were determined by GC-MS based on concentration of *N*-benzyl benzamide using m-xylene as internal standard. Isolated yields are in parentheses. ^c^TBHP addition rate of 0.8 µL min^−1^. ^d^10 mol% NHPI. ^e^Molecular sieves as additives (200 mg) and air. ^f^No catalyst. nd = not detected by GC-MS.

### *tert-*Butylhydroperoxide (TBHP) Decomposition

The rate of hydrolysis and subsequent formation of benzamide was increased with increasing concentration of the TBHP/water. To suppress the hydrolysis, TBHP in nonane (5.5 M, in molecular sieves) was used as the oxidant, minimizing the amount of water. Using TBHP in nonane, a maximum conversion of 10% and lower selectivity (15%) to imide (Entry 5, Table 1) was obtained. Negligible amounts of nonanol and nonanone were observed due to oxidation of nonane. To evaluate the rate of decomposition of TBHP, we probed the rate of decomposition of TBHP in the absence of amide substrate. Almost all of the TBHP was consumed within 45 min of reaction (Table S2). Alternatively, conjugated organic nitroxyl radicals combined with transition metal co-catalysts have been shown to be powerful catalytic systems for the auto-oxidation of hydrocarbons. Among those, N-hydroxyphthalimide (NHPI) is very popular in metal catalyzed oxidation of hydrocarbons^39^. Peroxyl radicals (ROO·) initiated by thermal decomposition of peroxides such as TBHP or deliberate introduction of free radical initiators, abstract an H-atom (>NO-H) from NHPI to form the PINO· radical (phthalimide *N*-oxyl)^40^. The combination of TBHP in nonane being added slowly (0.8 microliter/min) and using NHPI as an oxidation promoter (10 mol%) proved to be successful, as a significant increase in conversion (25%) was observed with high selectivity (90%) for imide (Entry 6, Table 1) was observed. Using molecular sieves as water scavengers, we surmised that adventitious water could be scavenged which would otherwise cause undesirable hydrolysis of imides. Thus the best conversion (90%) and selectivity (95%) towards imide were obtained (Entry 7, Table 1) with NHPI and molecular sieves.

Using air as oxidant decreased amide conversion (10%) significantly (Entry 8, Table 1) but showed the feasibility of using air as an oxidant. TBHP and NHPI without any catalyst did not produce any imide (Entry 12, Table 1); this signified the role of manganese oxide as catalyst in this reaction. Upon repeating the reaction under an argon atmosphere, we did not detect any imide, which proved the role of O_2_ as oxidant (Entry 9, Table 1). These results demonstrate the effectiveness of manganese oxides as heterogeneous catalysts for oxidation of amides to imides. The isolated yield (Entry 7) is in good agreement with the yield from GC-MS methods.

The oxidation of *N*-benzylbenzamide using Cs/Mn_2_O_3_ was then compared to different active, well known manganese oxide catalysts. Meso Mn_2_O_3_ having no Cs promoter ions showed much lower selectivity towards imide (Entries 1–2, Table 2). Cation vacancies in these oxides are the binding sites for amides based on our prior work where^35^ small loading (0.16% by weight) of Cs in the meso Cs/Mn_2_O_3_ material was critical for oxidation of amines to imines (which was used in this study). Using potassium containing manganese oxide octahedral molecular sieves (prepared by solvent free methods, K-OMS-2-SF) having similar Cs loading (0.16%), conversion and selectivity for imide decreased (Entry 4, Table 2). On the other hand, amorphous manganese oxide (AMO) prepared by redox methods displayed high conversion and selectivity (Entry 5, Table 2) to imides. Commercial manganese oxide was able to achieve minimal conversion without preferential selectivity towards the imide (Entry 6, Table 2). The reaction revealed no conversion in the absence of a catalyst (Entry 7, Table 2).

**Table 2:** Catalyst Scope for oxidation of *N*-benzylbenzamide.

Entry	Catalyst	Conv. (%)^b^	Selectivity (%)^b^	
			Diphenyl Imide	*N*-benzamide
1^c^	K-OMS-2	45	60	40
2^c^	Meso Mn_2_O_3_	50	60	40
3	Meso Cs/Mn_2_O_3_	77	67	33
4	Cs-K-OMS-2 (SF)	60	15	85
5	AMO (UCT-1)	60	60	40
6	C-MnO_2_	15	50	50
7	None	0	nd	nd

^a^Reaction conditions: *N*-benzylbenzamide (100 mg, 0.5 mmol, 1.0 equiv), catalyst (50 mg), acetonitrile (5 mL, 95 mmol, 190 equiv), TBHP (5 mmol, 0.45 g, 10 equiv) with addition rate of 0.8 µL min^−1^, NHPI (8 mg, 0.05 mmol, 0.1 equiv), molecular sieves (200 mg), 80 °C, 22 h. ^b^Conversions and selectivities were determined by GC-MS based on concentration of N-benzyl benzamide using m-xylene as internal standard. ^c^No NHPI was used. C-MnO_2_ denotes commercial manganese oxide. nd = not detected by GC-MS.

### Substrate Scope

The substrate scope and limitations were then explored for different types of amides. This methodology works well for oxidation of activated (e.g, benzylic CH_2_) α-CH_2_ groups with respect to amide. AMO was able to oxidize aromatic (Entries 1, 2, 4–7, Table 3), lactam (Entry 6, Table 3), and heteroaromatic (Entry 7, Table 3) amides to corresponding imides with excellent conversion and selectivity. No side reaction was detected in the case of a benzamide with a *para*-chloro substitution (Entry 2, Table 3). However, to our surprise with *ortho*-chloro substitution in the benzamide, no imide formation was detected (Entry 3, Table 3). Oxidation of 1-isoindoline (Entry 6, Table 3) was facile and produced a cyclic imide with excellent conversion (>99%) and selectivity (100%). The yields from GC-MS studies are in reasonable agreement with isolated yields. A thiophene amide was converted to the imide effectively (Entry 7, Table 3) without oxidizing the sulfur to sulfoxide or sulfone. We used α methyl-substituted *ortho* carboxylic derivative of benzyl amide to investigate if a product related to the initial stage of oxidation could be isolated (complete oxidation to the imide would be impossible). However, the substrate was totally inert (Entry 8, Table 3), which may be due to difficult abstraction of N-H hydrogen atoms due to a steric effect of the methyl group. Unactivated aliphatic amides (Entries 9 and 10, Table 3) were not converted to imides using this procedure. This may be rationalized by the strength of α-CH bonds in those substrates that falls outside the range of the NHPI derived PINO radical’s C-H activation scope.

**Table 3:** Oxidation of amides to imides by manganese oxides.

Entry	Substrate	Conv. (%)^b^	Selectivity (%)^b^ (Imide)
1^c^		90 (85)	95
2		90 (79)	98
3		0	0
4		92	94
5		90	94
6^c^		>99 (61)	100
7		85	92
8		0	0
9		0	0
10		0	0

^a^Reaction conditions: Amide (0.5 mmol, 1.0 equiv), AMO (50 mg), acetonitrile (5 mL, 95 mmol, 190 equiv), TBHP (5 mmol, 0.45 g, 10 equiv) with addition rate of 0.8 µL min^−1^, NHPI (8 mg, 0.05 mmol, 0.1 equiv), molecular sieves (200 mg), 80 °C, 22 h. No NHPI was used for isolating the imides. ^b^Conversions and selectivities were determined by GC-MS based on concentration of amide and selectivity toward the imide product. ^c^Used Cs/Mn_2_O_3_ instead of AMO. All runs were repeated at least twice, with a standard deviation of below 10%. Isolated Yields are in Parentheses.

### Kinetic and Stability Studies

Time dependent experiments for the oxidation of N-benzyl benzamide were conducted using meso Cs/Mn_2_O_3_ to study reaction rates (Fig. S1a). Periodic sampling was undertaken, the catalyst was separated by filtration, and conversion and selectivity of the filtrate were determined by GC-MS. Two separate time dependent experiments with and without NHPI were conducted. In the absence of NHPI, the reaction was very sluggish at the beginning (5% conversion after 8 h), whereas the presence of NHPI accelerated the reaction. Experiments with TBHP and NHPI indicated a first order rate equation with respect to amide (Fig. S1b) having a rate constant of 0.0025 min^−1^. PINO radical can abstract a hydrogen atom from amide (RCOCH_2_NH_2_) to initiate the reaction (Fig. S5). Since the addition rate of TBHP is slow, at the beginning the amount of TBHP was not enough for the generation of PINO radical. PINO radical reacts much faster than other peroxide based radicals with hydrocarbons, as confirmed by relative rate constants^6^. The catalyst stability was verified by performing reusability studies (Fig. S2b) with meso Cs/Mn_2_O_3_. After reaction, the catalyst was filtered, washed with excess solvent and water, dried under vacuum and reactivated at 250 °C for 30 min. No apparent loss of activity was observed after 3 catalytic cycles. Moreover, from the powder X-ray diffraction studies (Fig. S2a), it is apparent that the amorphous nature of the catalyst is retained after multiple reuses.

### Possible Reaction Pathway

**Figure 1:**
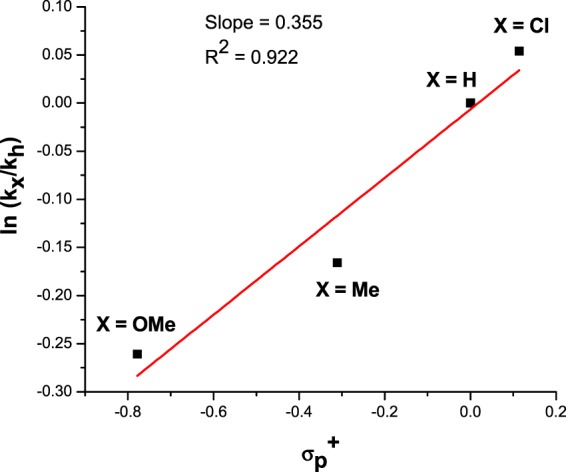
Hammett plot of competitive oxidation of para substituted *N*-benzylbenzamide. Reaction conditions are as described in Table 3 for 8 h. A linear relationship between ln(k_x_/k_H_) and Brown–Okamoto constant (σ_p_^+^) for para substituted benzylamines with slope (ρ) of 0.355 was obtained, which indicates the formation of a negatively charged transition state.

We investigated the relative rates of oxidation of para substituted *N*-benzylbenzamides (p-Cl, p-H, p-Me, p-OMe) to determine the electronic effect of the substituents. The Hammett equation can be used to interpret the electronic or steric influence of the substituents on the reaction intermediates. A linear relationship was found between ln (k_X_/k_H_) and the Brown–Okamato constant (σ_p_^+^) (Fig. 1). The slope of the plot resulted in a reaction constant (ρ) value of 0.355, which signified involvement of a partial negative charge at the reaction center in the transition state of the rate-limiting step (polar effects in similar radical reactions are known)^6^.

**Figure 2:**
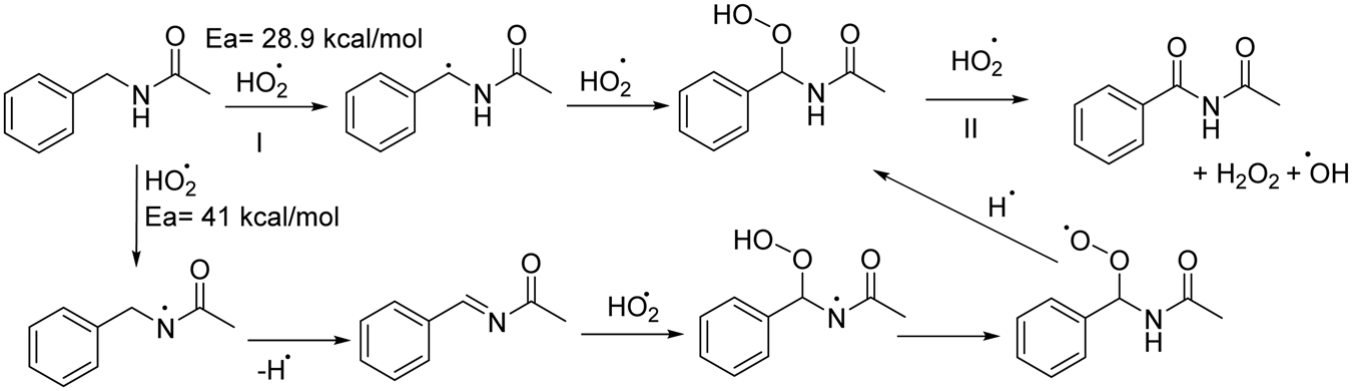
The C-H abstractions vs N-H abstraction pathways.

We propose a series of steps that can contribute to the formation of imides by manganese oxide (Fig. S3). Adsorbed amide molecules transfer an electron to the Mn center, followed by a cleavage of α C-H bond mediated by the NHPI/MnO_x_ system (Fig. S3). The oxidation of an alpha substituted amide is almost undetectable under the present reaction conditions, where the presence of the methyl group could have hindered the binding of amide, thus blocking the cleavage of the α C-H bond. The reaction pathway in theory may involve an imine intermediate, by further removal of proton and electron transfer to Mn atoms (Fig. 2). However according to the DFT calculation performed at MO6-2X for this reaction in the gas phase, this N-H hydrogen atom abstraction will have a 12 kcal/mol higher activation energy (Ea) barrier than α C-H atom abstraction. The reduction of Mn centers simultaneously generates the formation of labile lattice oxygen, which reoxidizes the Mn center with production of H_2_O_2_, which can easily be decomposed over manganese oxide and form water^1^. Once formed, the α hydroperoxide intermediate can readily undergo a further abstraction of the 2^nd^ benzylic hydrogen to yield the corresponding imide product. Hydroperoxide adducts were not detected by GC-MS, which signifies the rapid oxidation of the hydroperoxide species.

The aerobic oxidation pathway proceeds with meso Cs/Mn_2_O_3_ and AMO in the presence of NHPI. Perhaps a heterogeneous metal bound surface superoxide is formed as a first step^41–43^. This would then subsequently converted to a bound hydroperoxide followed by a similar mechanism TBHP as described in Fig. 2. This mechanism resembles the formylamide oxidation with peroxide and NHPI with a Co(II) catalyst. The exact mechanism is still under investigation with an emphasis on identification of all reactive oxygen species and potential correlation to reaction rates.

One of the major obstacles observed for many heterogeneous catalyst systems is of the product not desorbing from the catalyst surface. We wanted to study if there was a possibility that this phenomenon was occurring for the *N*-benzylbenzamide oxidation reaction; so, additional pre- and post-reaction characterizations were performed. X-ray Fluorescence spectroscopy (Table S3) was also done in order to further quantify the amount of product absorbed utilizing the most optimized conditions (Entry 1, Table 3). We were able to determine that there was approximately 5% by mass of the product still absorbed on the catalyst. These additional characterizations allowed us to partially account for the gaps between GC-MS and isolated yields.

## Conclusion

In summary, manganese oxide materials can catalyze the oxidation of aromatic substituted amides to imides. Among the several materials studied, mesoporous Cs promoted manganese oxide (meso Cs/Mn_2_O_3_) and amorphous manganese oxide (AMO) were shown to be the best catalysts. AMO oxidized a diverse array of aromatic amide derivatives to produce imides with high conversions and selectivities (90–100%). Although aerobic oxidation with meso Cs/Mn_2_O_3_ mediated by NHPI gave modest yields of imide, in the optimal procedure TBHP was used as oxidant. This manganese oxide mediated selective partial oxidation method is useful as compared to precious metal catalyst (e.g., Pt, Rh, Pd or Ir) that are used for partial oxidation in terms of activity, cost, and environmental impact.

## Methods

### Synthesis of manganese oxide materials

The manganese oxide materials were synthesized by well-established methods meso Cs/Mn_2_O_3_^17^, meso Mn_2_O_3_^17^, OMS-2 (SF)^44^ and AMO^45^.

### Synthesis of meso Cs/Mn_2_O_3_

Manganese (II) nitrate tetrahydrate (0.02 mol) was dissolved in a 1-butanol solution containing 0.188 mol (14 g) 1-butanol, 0.032 mol (2 g) of HNO_3_ and 3.4 × 10^−4^ mol (2 g) of P_123_ surfactant in a 150 mL beaker at RT and under magnetic stirring^17^. To this clear aqueous solution, 100 mL of 2.0 M CsNO_3_ was added maintaining the Mn:X ratio as 100:1. The resulting clear solution was then kept in an oven at 120 C for 3 h under air. The resulting black powder was washed with excess ethanol, centrifuged and dried in vacuum oven overnight. The dried black powder was then heated to 150 C for 12 h and 250 C for 3 h under air. The powder diffraction pattern of the material at this calcination temperature appears amorphous. However, upon heating at 450 °C or higher the XRD peak of this materials starts to appear crystalline with a pattern of Mn_2_O_3_ (Bixbyite)^17^. Meso Mn_2_O_3_was prepared using the same procedure without having the Cs ions.

### Synthesis of OMS-2(SF)

In a typical experiment, 9.48 g (0.06 mol) of KMnO_4_ and 22.05 g (0.09 mol) of Mn(Ac)_2_‚4H_2_O powders were mixed and ground homogeneously in a mortar^44^. The mixed powders were then placed in an autoclave and heated at 80 °C for 4 h. The resulting black product was thoroughly washed with excess deionized water, and finally dried at 80 °C in air overnight.

### Synthesis of AMO

A 60 mL volume of a 1.6 M Potassium permanganate solution was added dropwise to 100 mL of a 0.25 M Oxalic acid solution^45^. The mixture was stirred at room temperature for 2 hours. The resulting mixture was filtered and then washed with excess deionized water. The sample was then dried overnight at 90 °C.

### General procedure for oxidation of amides to imides

In a typical amide oxidation reaction, a mixture of *N*-benzylbenzamide (0.5 mmol, 100 mg), catalyst (50 mg), *N*-hydroxyphthalimide (10 mol%), molecular sieves (4A) and acetonitrile (5 mL) was added in a 50 mL two necked round bottom flask equipped with a condenser. A solution of tert-butylhydroperoxide (TBHP, 10 equiv) in nonane (5.5 M) was added dropwise with a rate of 0.8 microliter min^−1^. The reaction mixture was heated to reflux under vigorous stirring (700 rpm) until the full consumption of TBHP. After reaction, the mixture was cooled and the catalyst was removed by filtration. The product analysis was done using GC-MS (gas chromatography-mass spectrometry). The conversion was determined based on the concentration of amides. Most reactions were repeated twice, and the average values were used. The imide products were isolated by silica gel column chromatography using hexane and (30–50%) ethyl acetate as the eluent and identified by ^1^H and ^13^C NMR spectra.

### Characterization

The GC-MS analyses were performed with a 7820A GC system connected with a mass detector of 5975 series MSD from Agilent Technologies and a nonpolar cross-linked methyl siloxane column with dimensions of 12 in × 0.200 mm × 0.33 µm was used. The ^1^H and ^13^C NMR spectra were recorded on a Bruker AVANCE III- 400 MHz spectrometer. ^1^H NMR spectra were collected at 400 MHz with chemical shift referenced to the residual CHCl_3_ peak in CDCl_3_ (δ: H 7.26 ppm). ^13^C NMR spectra were collected at 100 MHz and referenced to the CDCl_3_ signal (δ: C 77.0 ppm)^46^. Only in case of phthalimide the solvent was DMSO-d6, and chemical shifts were referenced to the residual DMSO-d5 peak in DMSO-d6 (δ: H 2.50 ppm) for ^1^H NMR and the DMSO-d6 peak (δ: C 39.51 ppm) for ^13^C NMR^46^. The spectral data of imide products were compared with the literature reports^47^.

## Electronic supplementary material

Supplementary Information

## References

[1] Vanderwal CD, Jacobsen EN (2004). Enantioselective Formal Hydration of α,β-Unsaturated Imides by Al-Catalyzed Conjugate Addition of Oxime Nucleophiles. J. Am. Chem. Soc..

[2] Balskus EP, Jacobsen EN (2006). α,β-Unsaturated β-Silyl Imide Substrates for Catalytic, Enantioselective Conjugate Additions:  A Total Synthesis of (+)-Lactacystin and the Discovery of a New Proteasome Inhibitor. J. Am. Chem. Soc..

[3] Ali MA, Siddiki SMAH, Kon K, Hasegawa J, Shimizu K (2014). Versatile and Sustainable Synthesis of Cyclic Imides from Dicarboxylic Acids and Amines by Nb2O5 as a Base-Tolerant Heterogeneous Lewis Acid Catalyst. Chem. – A Eur. J..

[4] Nicolaou KC, Mathison CJN (2005). Synthesis of Imides, N-Acyl Vinylogous Carbamates and Ureas, and Nitriles by Oxidation of Amides and Amines with Dess–Martin Periodinane. Angew. Chemie Int. Ed..

[5] De Sarkar S, Ackermann L (2014). Ruthenium(II)-Catalyzed C-H Activation with Isocyanates: A Versatile Route to Phthalimides. Chem. – A Eur. J..

[6] Minisci F, Punta C, Recupero F, Fontana F, Pedulli GF (2002). Aerobic Oxidation of N-Alkylamides Catalyzed by N-Hydroxyphthalimide under Mild Conditions. Polar and Enthalpic Effects. J. Org. Chem..

[7] Bietti M (2016). Kinetic Study of the Reaction of the Phthalimide-N-oxyl Radical with Amides: Structural and Medium Effects on the Hydrogen Atom Transfer Reactivity and Selectivity. J. Org. Chem..

[8] Taherpour AA, Abramian A, Kardanyazd H (2007). Synthesis of Imide by Oxidation of N-Alkyl Amides under Microwave Irradiation. Chinese J. Org. Chem..

[9] Gupta, R. B. *Hydrogen Fuel: Production, Transport, and Storage*. (CRC Press, 2009).

[10] Brock SL (1998). A Review of Porous Manganese Oxide Materials. Chem. Mater..

[11] Suib SL (2008). Porous Manganese Oxide Octahedral Molecular Sieves and Octahedral Layered Materials. Acc. Chem. Res..

[12] Tian, Z.-R. *et al*. Manganese Oxide Mesoporous Structures: Mixed-Valent Semiconducting Catalysts. *Science (80-.)*. **276**, 926 LP-930 (1997).

[13] Post JE (1999). Manganese oxide minerals: Crystal structures and economic and environmental significance. Proc. Natl. Acad. Sci..

[14] Kim SC, Shim WG (2010). Catalytic combustion of VOCs over a series of manganese oxide catalysts. Appl. Catal. B Environ..

[15] Nicolas-Tolentino E, Tian Z-R, Zhou H, Xia G, Suib SL (1999). Effects of Cu2+ Ions on the Structure and Reactivity of Todorokite- and Cryptomelane-Type Manganese Oxide Octahedral Molecular Sieves. Chem. Mater..

[16] Poyraz AS (2013). Bimodification of Mesoporous Silicon Oxide by Coupled ‘In Situ Oxidation at the Interface and Ion Exchange’ and its Catalytic Activity in the Gas-Phase Toluene Oxidation. ChemCatChem.

[17] Biswas S (2015). Ion induced promotion of activity enhancement of mesoporous manganese oxides for aerobic oxidation reactions. Appl. Catal. B Environ..

[18] Santos VP, Pereira MFR, Órfão JJM, Figueiredo JL (2009). Catalytic oxidation of ethyl acetate over a cesium modified cryptomelane catalyst. Appl. Catal. B Environ..

[19] Son Y-C, Makwana VD, Howell AR, Suib SL (2001). Efficient, Catalytic, Aerobic Oxidation of Alcohols with Octahedral Molecular Sieves. Angew. Chemie.

[20] Opembe NN, Son Y-C, Sriskandakumar T, Suib SL (2008). Kinetics and Mechanism of 9H-Fluorene Oxidation Catalyzed by Manganese Oxide Octahedral Molecular Sieves. ChemSusChem.

[21] Ghosh†, R. *et al*. Role of Manganese Oxide Octahedral Molecular Sieves in Styrene Epoxidation, 10.1021/JP056961N (2006). 10.1021/jp056961n 16599543

[22] Wang Y, Kobayashi H, Yamaguchi K, Mizuno N (2012). Manganese oxide-catalyzed transformation of primary amines to primary amides through the sequence of oxidative dehydrogenation and successive hydration. Chem. Commun..

[23] Yamaguchi K, Kobayashi H, Oishi T, Mizuno N (2012). Heterogeneously Catalyzed Synthesis of Primary Amides Directly from Primary Alcohols and Aqueous Ammonia. Angew. Chemie Int. Ed..

[24] Yamaguchi K (2013). Green oxidative synthesis of primary amides from primary alcohols or aldehydes catalyzed by a cryptomelane-type manganese oxide-based octahedral molecular sieve, OMS-2. 318 Catal. Sci. Technol. Catal. Sci. Technol.

[25] Wang Y, Yamaguchi K, Mizuno N (2012). Manganese Oxide Promoted Liquid-Phase Aerobic Oxidative Amidation of Methylarenes to Monoamides Using Ammonia Surrogates. Angew. Chemie.

[26] Yamaguchi K, Wang Y, Mizuno N (2012). Manganese Oxide-catalyzed Additive- and Solvent-free Aerobic Oxidative Synthesis of Primary Amides from Primary Amines. Chem. Lett..

[27] Sithambaram S, Kumar R, Son Y, Suib S (2008). Tandem catalysis: Direct catalytic synthesis of imines from alcohols using manganese octahedral molecular sieves. J. Catal..

[28] Chen B (2014). Direct imine formation by oxidative coupling of alcohols and amines using supported manganese oxides under an air atmosphere. Green Chem..

[29] Zhang, Z. *et al*. tert-Butyl hydroperoxide (TBHP)-mediated oxidative self-coupling of amines to imines over a α-MnO 2 catalyst. 10.1039/c3gc42312c.

[30] Jin X (2012). Heterogeneously catalyzed selective aerobic oxidative cross-coupling of terminal alkynes and amides with simple copper(ii) hydroxide. Chem. Commun..

[31] Yamaguchi K, Wang Y, Oishi T, Kuroda Y, Mizuno N (2013). Heterogeneously Catalyzed Aerobic Cross-Dehydrogenative Coupling of Terminal Alkynes and Monohydrosilanes by Gold Supported on OMS-2. Angew. Chemie Int. Ed..

[32] Jin, X., Yamaguchi, K. & Mizuno, N. Aerobic cross-dehydrogenative coupling of terminal alkynes and tertiary amines by a combined catalyst of Zn 2+ and OMo-2. 10.1039/c4ra05105j.

[33] Yu H, Chen Y, Zhang Y (2015). TBHP/TEMPO-Mediated Oxidative Synthesis of Imides from Amides. Chinese J. Chem..

[34] Poyraz AS, Kuo C-H, Biswas S, King’ondu CK, Suib SL (2013). A general approach to crystalline and monomodal pore size mesoporous materials. Nat. Commun..

[35] Biswas S (2015). Aerobic Oxidation of Amines to Imines by Cesium-Promoted Mesoporous Manganese Oxide. ACS Catal..

[36] Dutta B (2016). Mesoporous Manganese Oxide Catalyzed Aerobic Oxidative Coupling of Anilines To Aromatic Azo Compounds. Angew. Chemie Int. Ed..

[37] Tang R-Y (2011). TBHP-mediated oxidative thiolation of an sp3 C–H bond adjacent to a nitrogen atom in an amide. Chem. Commun..

[38] Grootboom N, Nyokong T (2002). Iron perchlorophthalocyanine and tetrasulfophthalocyanine catalyzed oxidation of cyclohexane using hydrogen peroxide, chloroperoxybenzoic acid and tert-butylhydroperoxide as oxidants. J. Mol. Catal. A Chem..

[39] Sheldon RA, Arends IWCE (2004). Organocatalytic Oxidations Mediated by Nitroxyl Radicals. Adv. Synth. Catal..

[40] Hermans, I. *et al*. Mechanism of the catalytic oxidation of hydrocarbons by N-hydroxyphthalimide: a theoretical studyElectronic supplementary information (ESI)available: all discussed TS and important intermediates (geometries, energies, ZPE, rotational constants and frequencies). *Chem. Commun*. o**5**, 1140, http://www.rsc.org/suppdata/cc/b4/b401050g/ (2004). 10.1039/b401050g 15116224

[41] Ishii Y, Iwahama T, Sakaguchi S, Nakayama K, Nishiyama Y (1996). Alkane Oxidation with Molecular Oxygen Using a New Efficient Catalytic System: N-Hydroxyphthalimide (NHPI) Combined with Co(acac)(n)() (n = 2 or 3). J. Org. Chem..

[42] Ishii Y, Sakaguchi S (2006). Recent progress in aerobic oxidation of hydrocarbons by N-hydroxyimides. Catal. Today.

[43] Shibamoto A, Sakaguchi S, Ishii Y (2000). Aerobic Oxidation of Methylpyridines to Pyridinecarboxylic Acids Catalyzed by N -Hydroxyphthalimide. Org. Process Res. Dev..

[44] Ding YS (2005). Synthesis and catalytic activity of cryptomelane-type manganese dioxide nanomaterials produced by a novel solvent-free method. Chem. Mater..

[45] Cao H, Suib SL (1994). Highly efficient heterogeneous photooxidation of 2-propanol to acetone with amorphous manganese oxide catalysts. J. Am. Chem. Soc..

[46] Gottlieb HE, Kotlyar V, Nudelman A (1997). NMR chemical shifts of common laboratory solvents as trace impurities. J. Org. Chem..

[47] Evans DA, Nagorny P, Xu R (2006). Ceric ammonium nitrate promoted oxidation of oxazoles. Org. Lett..

