# Supplementary material for: Heterogeneous Catalytic Oxidation of Amides to Imides by Manganese Oxides
Source: Sci Rep. 2018 Sep 11;8:13649. doi: 10.1038/s41598-018-31729-3 (PMC6134084; doi:10.1038/s41598-018-31729-3)
Supplement: Supplementary file 1 — Supplementary Information [file 41598_2018_31729_MOESM1_ESM.docx]

**Supporting Information for:**

Heterogeneous Catalytic Oxidation of Amides to Imides by Manganese Oxides

Sourav Biswas^1^, Harshul Khanna^2^, Quddus A. Nizami^3^, Donald R. Caldwell^1^, Katherine T. Cavanaugh^1^ , Amy R. Howell^1^, Sumathy Raman^3^, Steven L. Suib^1,2^*, and Partha Nandi^3^*

^1^Department of Chemistry, University of Connecticut, Storrs, CT 06269 (USA)

^2^Institute of Materials Science, University of Connecticut, Storrs, CT 06269 (USA)

^3^Corporate Strategic Research, ExxonMobil Research and Engineering Company, 1545 US 22 East, Annandale, NJ 08801 (USA)

*Correspondence and requests for materials should be addressed to S.L.S. (steven.suib@uconn.edu) and P. N. (partha.nandi@exxonmobil.com)

**Table of Contents**

**I. Table S1. Optimization of oxidation of *N*-benzyl benzamide……...…….…………...……..…S1**

**II. Table S2. Decomposition of TBHP by meso Cs/MnOx…..............................................S2**

**III.** **Figure S1. Time dependent study of oxidation of amides by meso Cs/MnO_x_ and corresponding kinetic plot .…...…………….…………………………...…………………………….…..................................S2**

**IV. Figure S2. Reusability Study for Meso Mn_2_O_3_ for Amide Oxidation …………………………………S3**

**V. Figure S3.** **Proposed mechanism of MnOx-NHPI mediated oxidation of *N*-benzyl benzamide………………………………………………………………………………...................................**.**..S4**

**VI. Figure S4. Hydrolysis of imide to benzamide (side reaction)…………………………....……S4**

**VII. Procedure of preparing *N*-((thiophen-2 yl)methyl)benzamide……………..…………….....S5**

**VIII. Procedure of preparing** ***N*-(2-chlorobenzyl)benzamide………….……………..…………….....S5**

**IX. Procedure of preparing** **4-chloro-*N*-(phenylmethyl)-benzamide……..…………….....S6**

**X. Table S3.** **XRF of catalyst Pre and post oxidation N-benzyl benzamide reaction...............**.**..S6**

**XI. Mass spectra of imides……………………………...………..……….…………………………...…….......S7**

**XII. NMR spectra of N-((thiophen-2-yl)methyl)benzamide.………………………………………….…S10**

**XIII. NMR spectra of N-(2-chlorobenzyl)benzamide…………………………………………………….…S12**

**XIV. NMR spectra of 4-chloro-*N*-(phenylmethyl)-benzamide ………………………………………….…S14**

**XV. NMR spectra of imides……………..……………………………….…………………………………...…....S16**

Table S1. Screening of TBHP/catalyst loading and different solvents of N-benzyl benzamide oxidation by meso Cs/MnOx ^a^

| Entry | Solvent | Catalyst (mg) | TBHP (mmol) | Conv^b^ (%) | Selectivity^b^ (%) | | |
| --- | --- | --- | --- | --- | --- | --- | --- |
|  |  |  |  |  | 1a | 2a | 3a |
| 1 | ACN | 50 | 0 | 0 | nd | nd | nd |
| 2 | ACN | 50 | 1.5 | 4 | 50 | 28 | 20 |
| 3 | ACN | 50 | 2 | 3 | 80 | 11 | 9 |
| 4 | ACN | 100 | 1 | 15 | 60 | 15 | 18 |
| 5 | Dioxane | 100 | 1 | 0 | nd | nd | nd |
| 6 | THF | 100 | 1 | 0 | nd | nd | nd |
| 7 | Toluene | 100 | 1 | 11 | 0 | 82 | 9 |
| 8 | CHCl_3_ | 100 | 1 | 20 | 95 | 4 | <1 |

[a] Reaction conditions: N-benzyl benzamide (105.5 mg, 0.5 mmol, 1.0 equiv), meso Cs/MnOx (50 -100 mg), 80°C for acetonitrile (5.0 mL, 95 mmol, 190 equiv) , 100°C for dioxane (5.0 mL, 56 mmol, 112 equiv), 66°C for THF (5.0 mL, 62 mmol, 124 equiv) and toluene (5.0 mL, 47 mmol, 94 equiv) 60°C for chloroform (5.0 mL, 58 mmol, 116 equiv) tert-butylhydroperoxide (TBHP, 70% in water), 22 h. [b] Determined by GC-MS based on concentration of N-benzyl benzamide. nd = not detected

Table S2. Decomposition of TBHP by meso Cs/MnOx ^a^

| Entry | Time (min) | Conversion ^b^ (%) |
| --- | --- | --- |
|  |  |  |
| 1 | 7.5 | 82 |
| 2 | 15 | 82 |
| 3 | 22.5 | 83 |
| 4 | 30 | 84 |
| 5 | 37.5 | 86 |
| 6 | 45 | >99 |

^a^ Reaction conditions: catalyst (50 mg), acetonitrile (5.0 mL), TBHP (5.5 M in nonane, 1 mL), 80 °C, 45 min. ^b^ Conversions were determined by GC-MS.

Figure S1 (a) Time dependent study of oxidation of amides by meso Cs/MnO_x_ and (b) corresponding kinetic plot, which signifies a first order reaction with respect to amide having a rate constant of 0.0025 min^-1^. N-benzyl benzamide (0.5 mmol), meso Cs/MnO_x_ (50 mg), acetonitrile (5.0 mL), TBHP (5.5 M in nonane) with addition rate of 0.8 µL min^-1^, NHPI (10 mol%), 22 h.


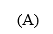

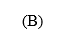

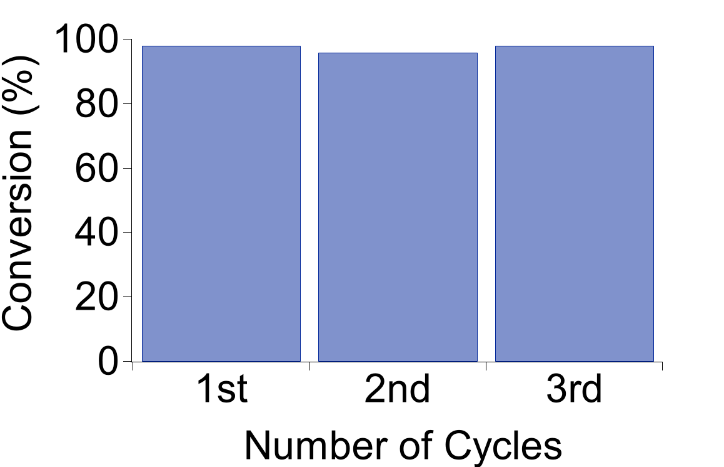


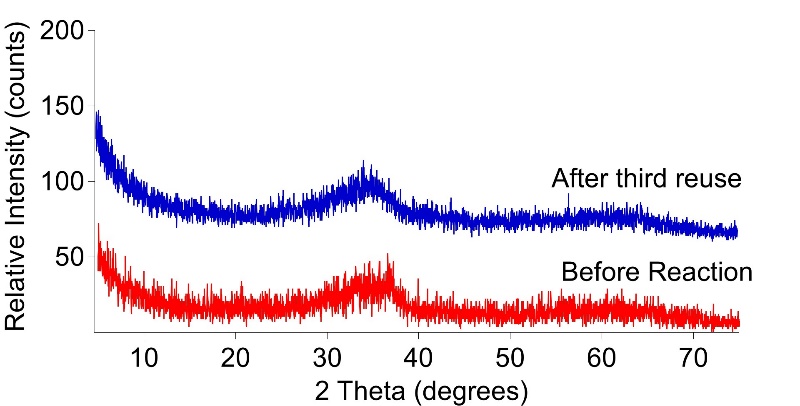


Figure S2. (A) PXRD of meso Mn_2_O_3_ before and after third reuse. No significant change was observed even after third reuse. (B) Reusability test of the catalyst. Reaction condition: N-benzylbenzamide (0.5 mmol, 1.0 equiv), meso Cs/ Mn_2_O_3_ (50 mg), acetonitrile (5 mL, 95 mmol, 190 equiv), TBHP (5 mmol, 0.45 g, 10 equiv) with addition rate of 0.8 µL min^-1^, NHPI (8.16 mg, 0.05 mmol, 0.1 equiv), molecular sieves (200 mg), 80 °C, 22 h.

Figure S3. Proposed mechanism of MnOx-NHPI mediated oxidation of N-benzyl benzamide.

Figure S4. Hydrolysis of imide to benzamide (side reaction).

**Procedure of preparing *N*-((thiophen-2-yl)methyl)benzamide**

In a typical synthesis, benzoyl chloride (3 mmol) was added dropwise in a solution containing (thiophen-2-yl)methanamine (3 mmol) and dry toluene (10 ml) in a 50 mL round bottom flask at 0°C (ice bath). The solution was stirred for 30 min at 0°C and then refluxed for 2 h at 100°C. The reaction mixture was cooled and filtered. Sodium bicarbonate was added to the filtrate and stirred for 1 h and filtered again. The product (white solid) was isolated by evaporating the solvent under reduced pressure and dried under vacuum. The product was identified by GC-MS and ^1^H and ^13^C NMR and molar mass was found to be 217 gmol^-1^.

**Procedure of preparing *N*-(2-chlorobenzyl)benzamide**^1^

In a typical synthesis, 2-chlorobenzylamine (3 mmol) was added dropwise in a solution containing benzoyl chloride (3 mmol), triethylamine (4.8 mmol) and dichloromethane (7 mL) in a 50 mL round bottom flask. The solution was stirred for 2 hours at room temperature. The solution was washed twice with 1M hydrochloric acid (5 mL) and then brine (5 mL). The organic layer was then treated with manganese. The product (white solid) was isolated by evaporating the solvent under reduced pressure and dried under vacuum. The product was identified by GC-MS and ^1^H and ^13^C NMR and molar mass was found to be 245 gmol^-1^. The solution was concentrated to give a crude product, which was purified by flash column chromatography on silica gel (80:20 hexanes: EtOAc) to obtain *N*-(2-chlorobenzyl)-benzamide as a white solid: ^1^H NMR (400 MHz, CDCl_3_) δ 7.78 (d, 2H), 7.2-7.50 (m, 9H), 6.7 (s, 1H), 4.72 (d, 2H); ^13^C NMR (100 MHz, CDCl_3_) δ 167.4, 135.6, 134.3, 133.7, 131.6, 130.4, 129.0, 128.6, 127.2, 127.0, 42.1. Spectra were compared to literature.^2^

**Procedure of preparing 4-chloro-*N*-(phenylmethyl)-benzamide**^3^

In a typical synthesis, a solution of *p*-chlorobenzoic acid (5.3 mmol) and benzylamine (5.6 mmol) in 53 mL of CH_2_Cl_2_ was added to DIPEA (15.9 mmol). Then, HBTU (8.0 mmol) was then added at 0 ˚C. The resulting mixture was stirred at r.t. for 3 h. The reaction mixture was diluted with CH_2_Cl_2_ (420 mL) and washed with water (100 mL). The organic layer was separated and dried with anhydrous MgSO_4_. The solution was concentrated to give a crude product, which was purified by flash column chromatography on silica gel (80:20 hexanes:EtOAc) to obtain 4-chloro-*N*-(phenylmethyl)-benzamide (984 mg, 75%) as a white solid: ^1^H NMR (400 MHz, CDCl_3_) δ 7.72 (d, *J*= 8.0 Hz, 2H), 7.41 (d, *J*= 8.0 Hz, 2H), 7.40-7.29 (m, 5H), 6.35 (br s, 1H), 4.63 (d, *J*= 4.0 Hz, 2H); ^13^C NMR (100 MHz, CDCl_3_) δ 167.7, 138.3, 138.2, 133.1, 129.2, 128.7, 128.3, 128.1, 44.6

**
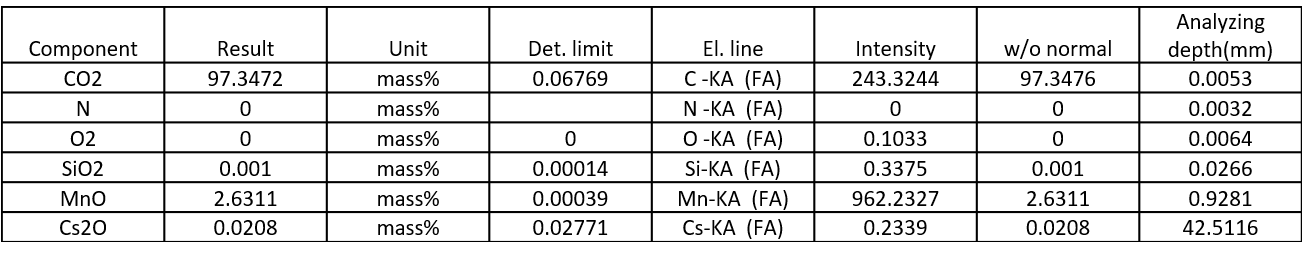
**

1. Pre-reaction

(B) Post-reaction

**
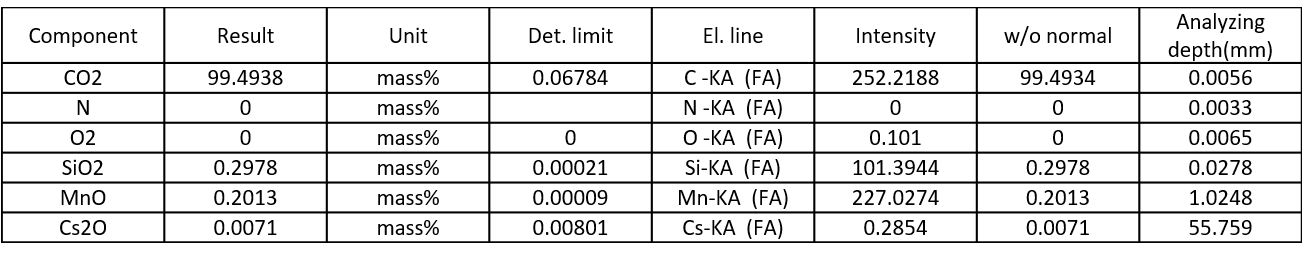
**

Table S3. XRF data (A) Pre-reaction and (B) Post-reaction condition: N-benzylbenzamide (0.5 mmol, 1.0 equiv), meso Cs/ Mn_2_O_3_ (50 mg), acetonitrile (5 mL, 95 mmol, 190 equiv), TBHP (5 mmol, 0.45 g, 10 equiv) with addition rate of 0.8 µL min^-1^, NHPI (8.16 mg, 0.05 mmol, 0.1 equiv), molecular sieves (200 mg), 80 °C, 22 h.

**Mass spectra of imides**


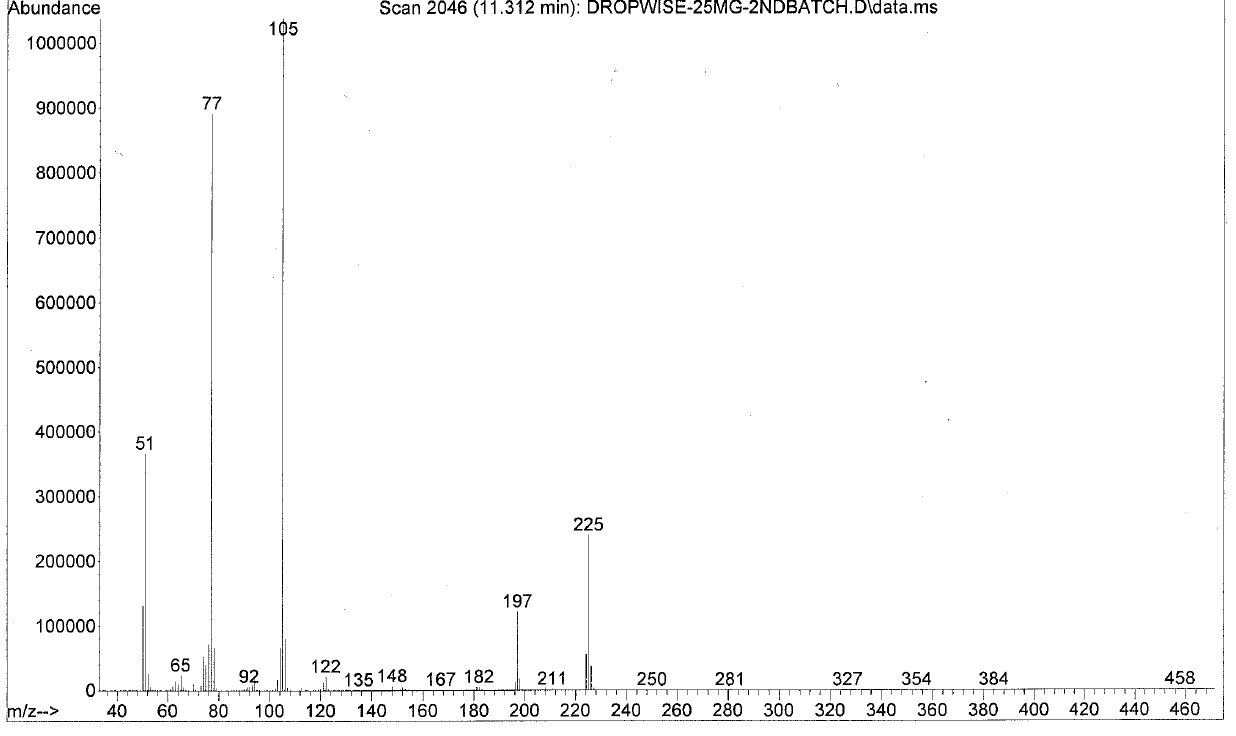


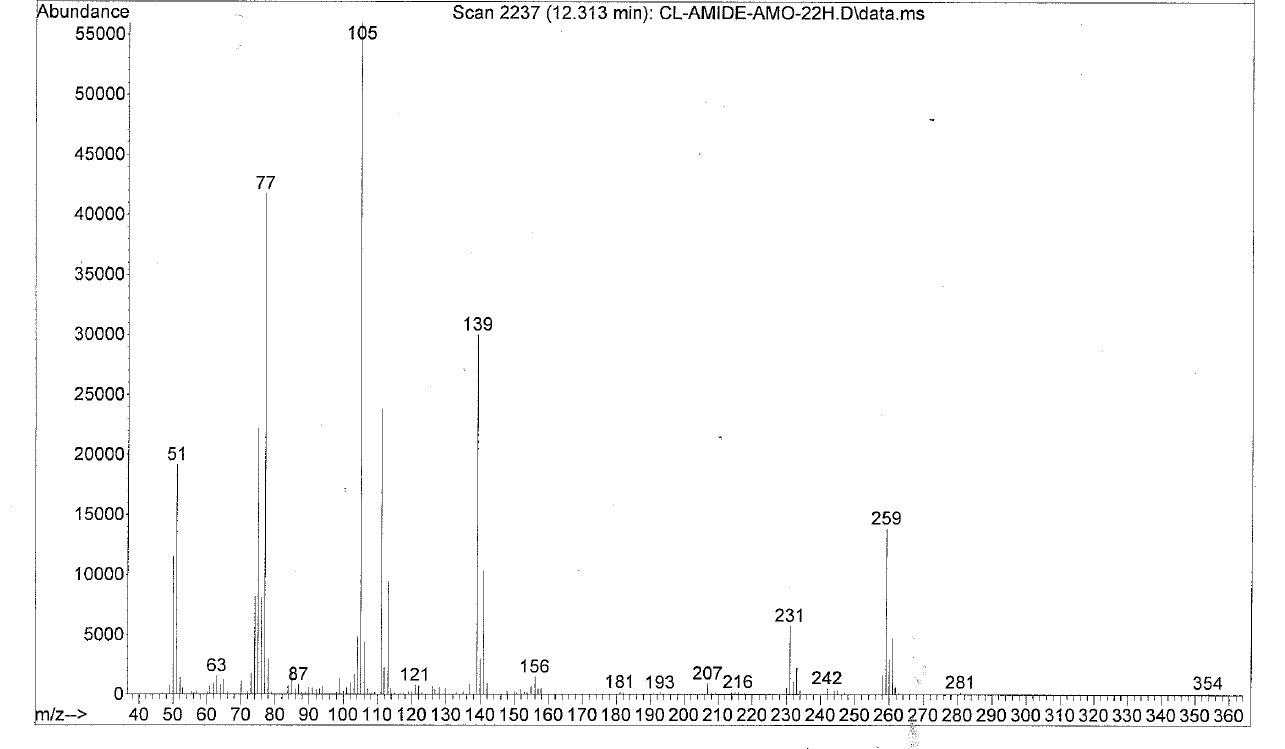


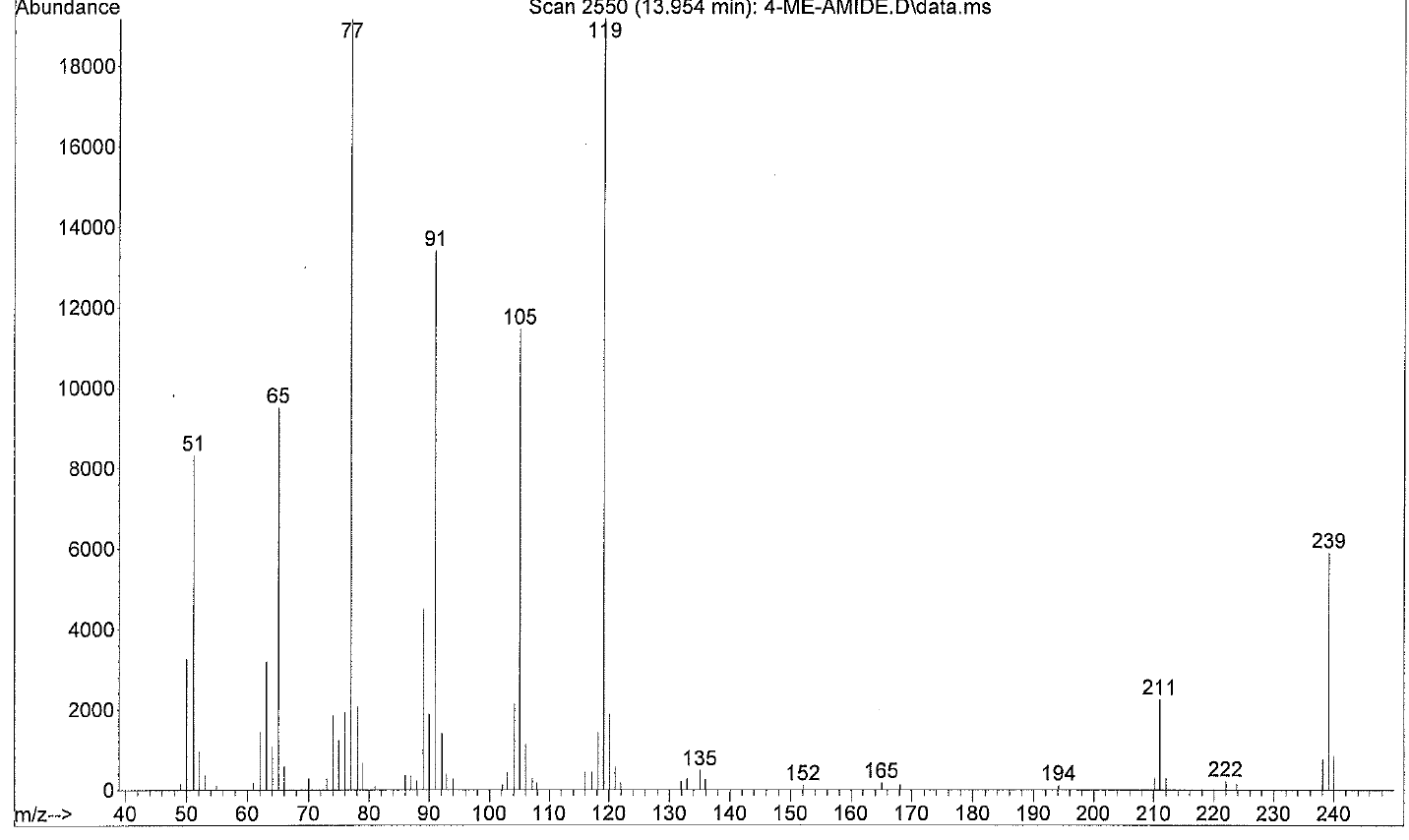

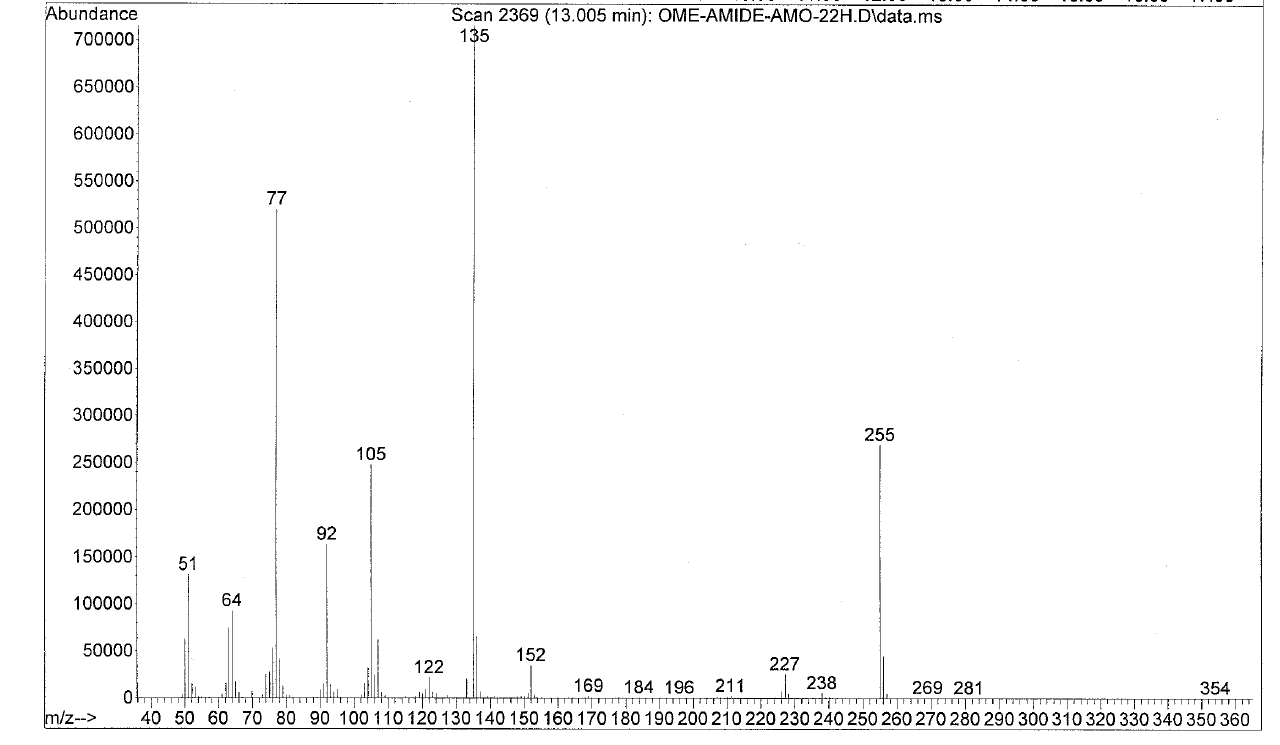

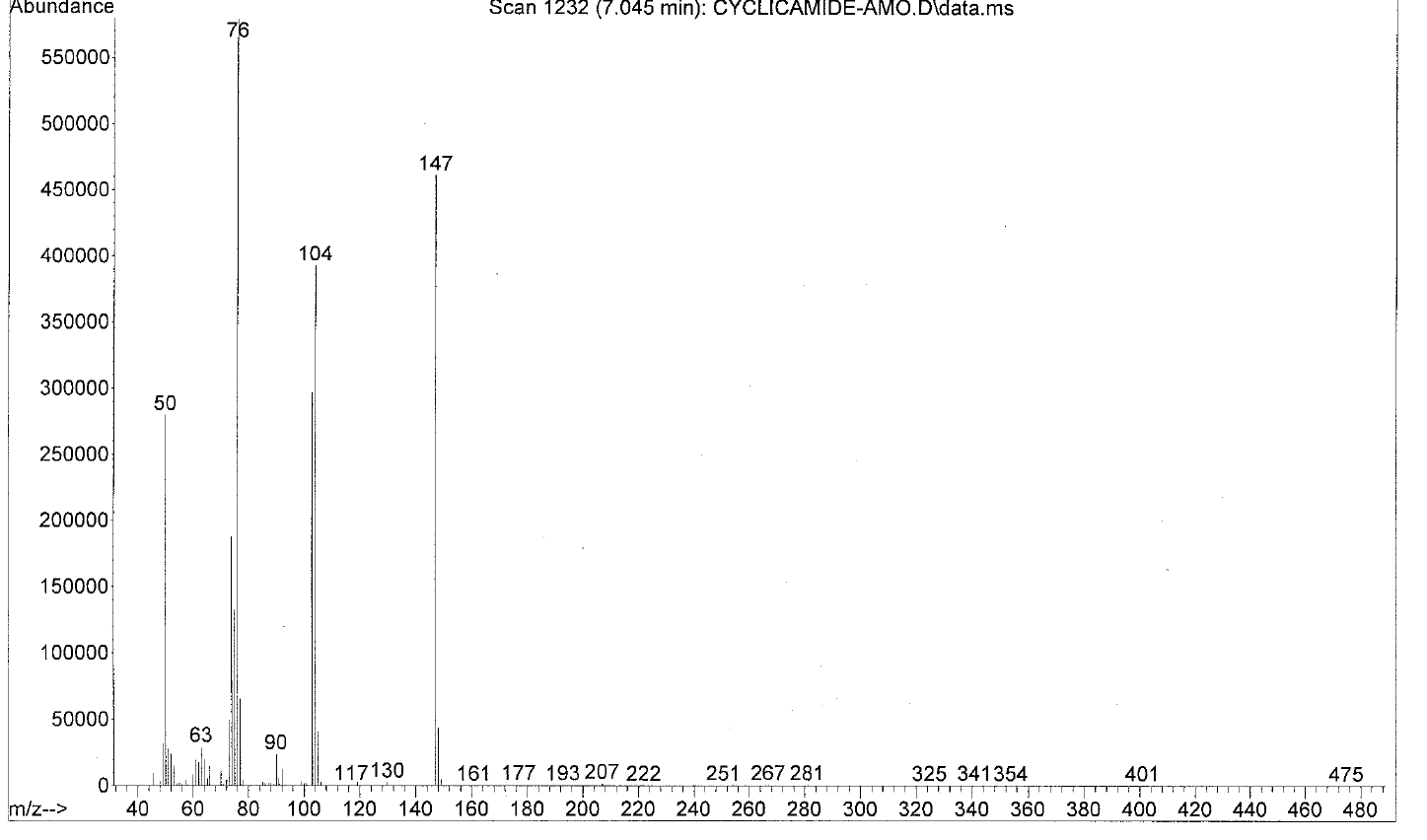

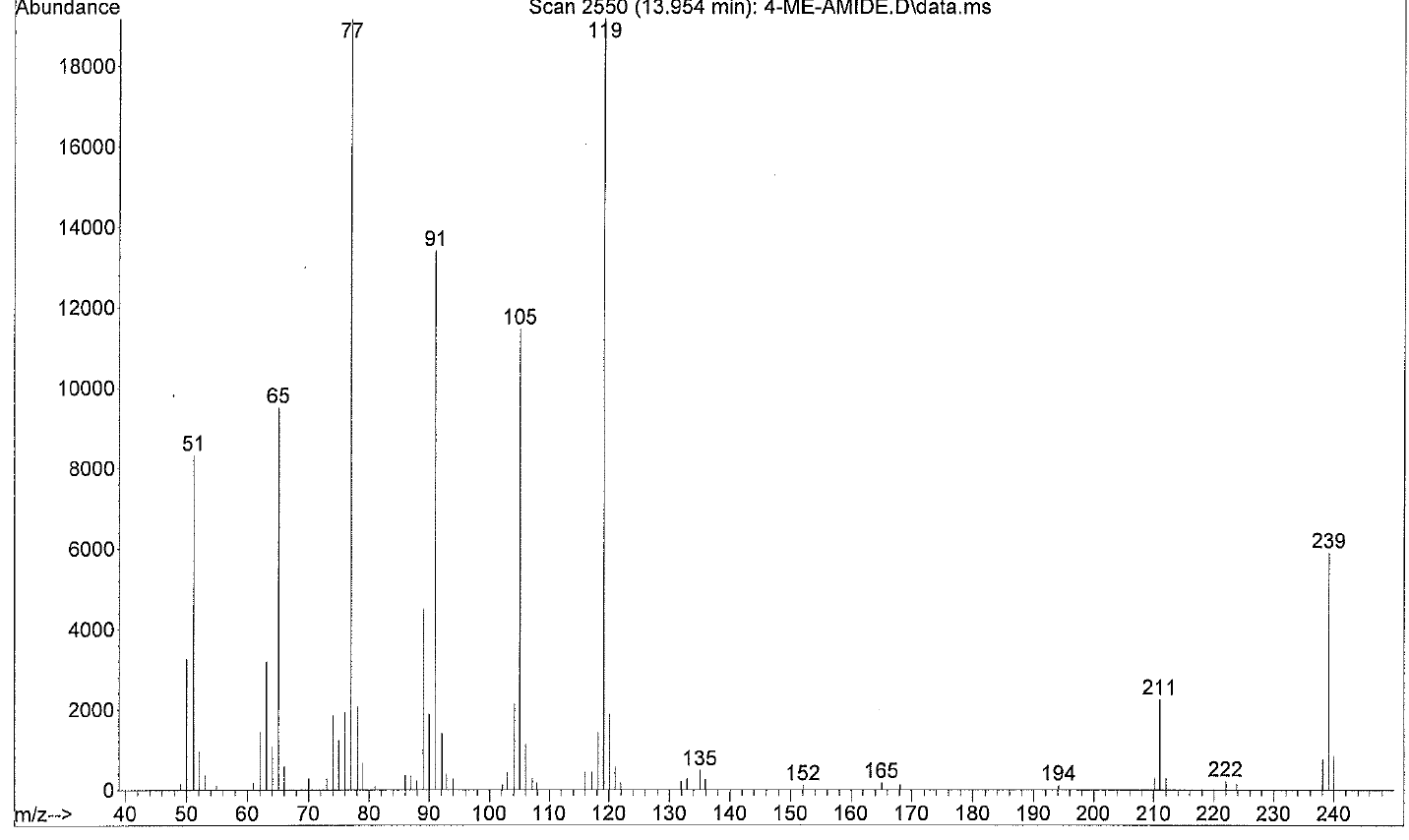

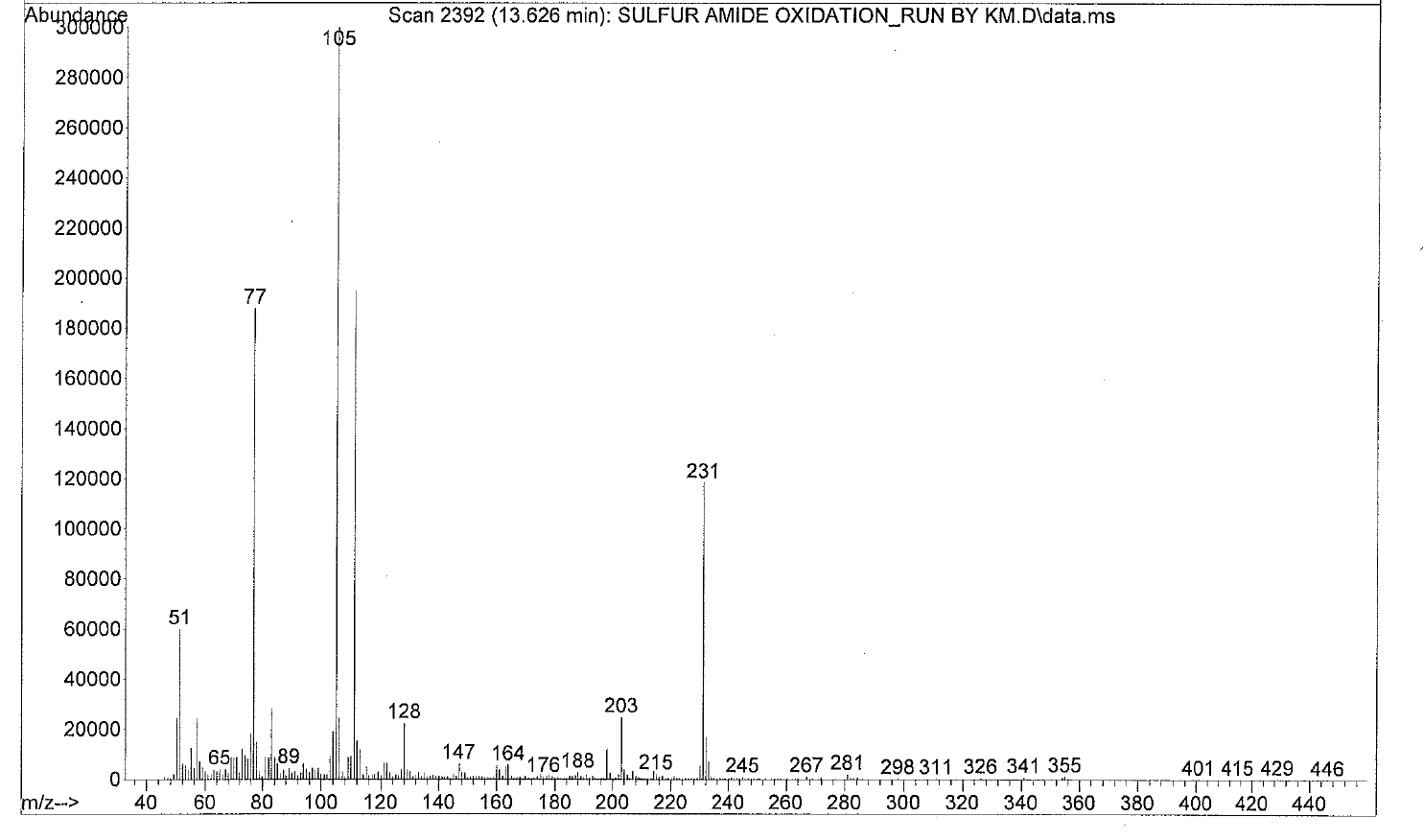


**NMR spectra of N-((thiophen-2-yl)methyl)benzamide**


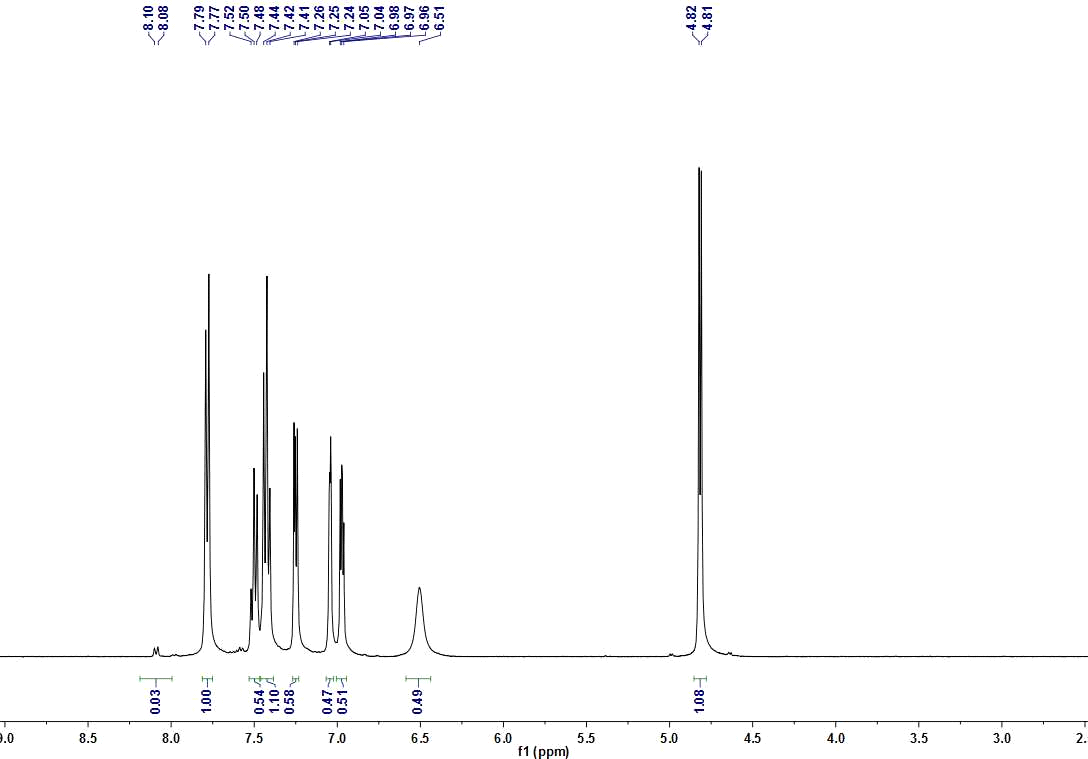

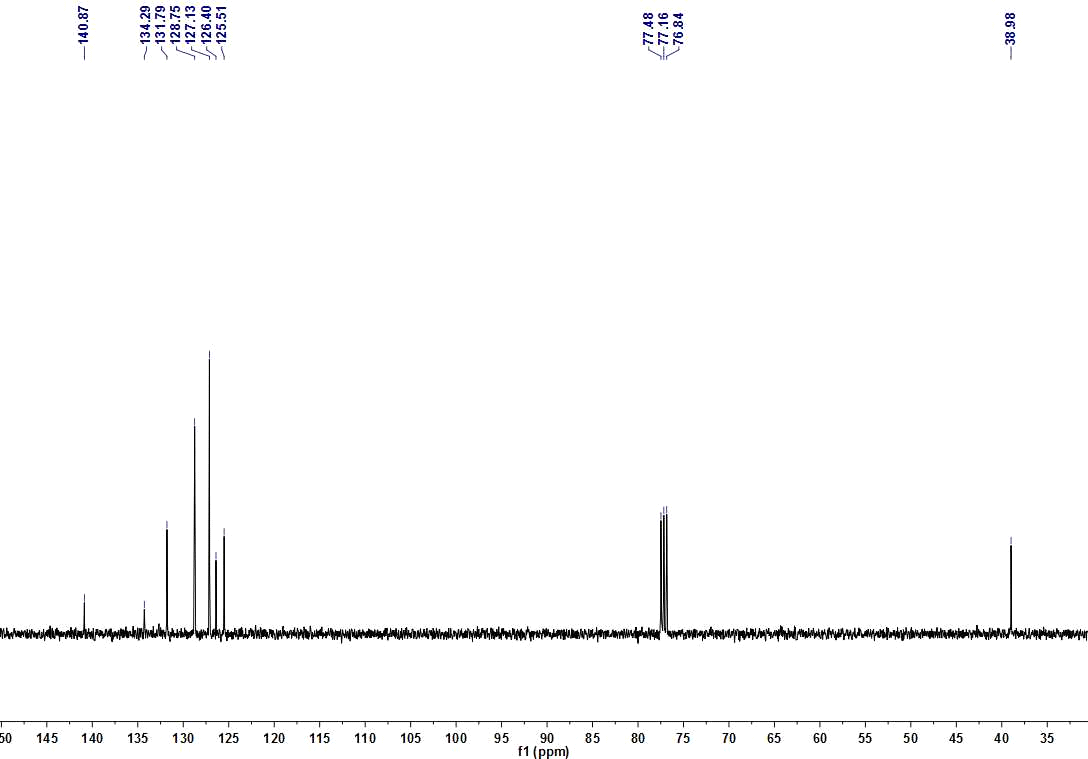


**NMR spectra of N-(2-chlorobenzyl)benzamide**


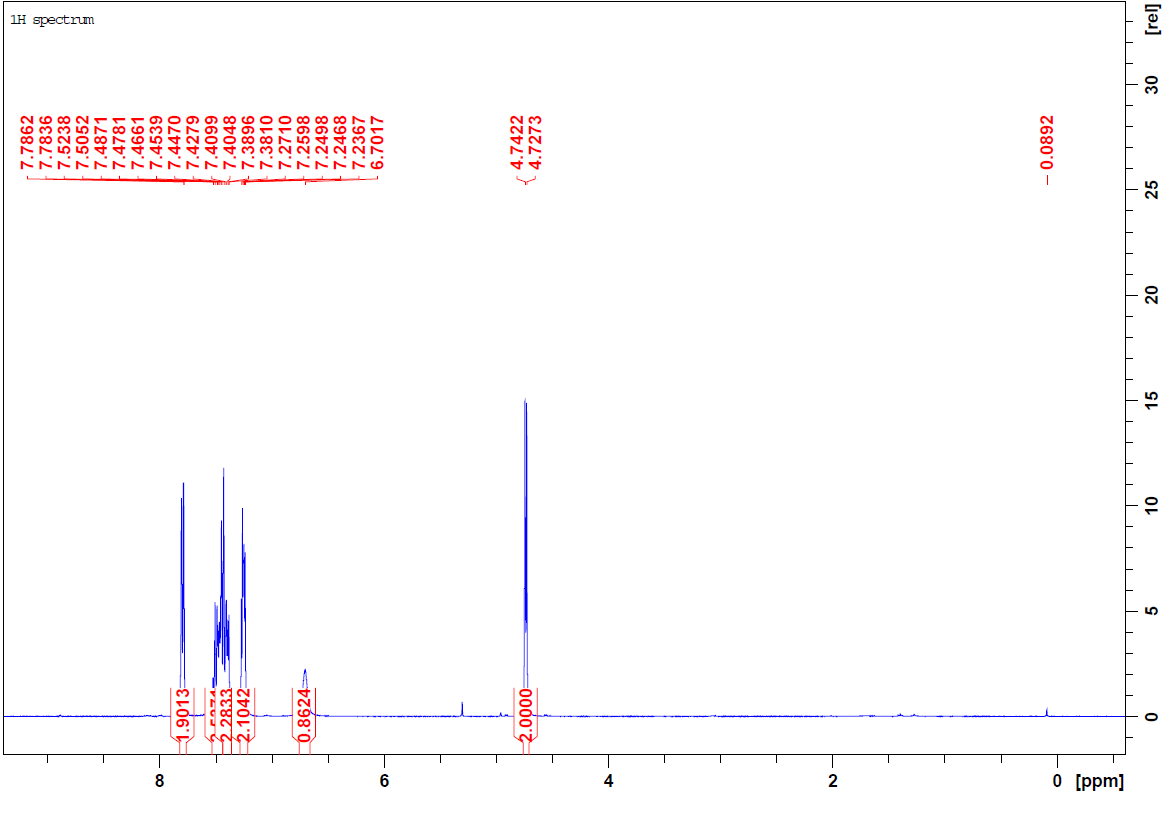


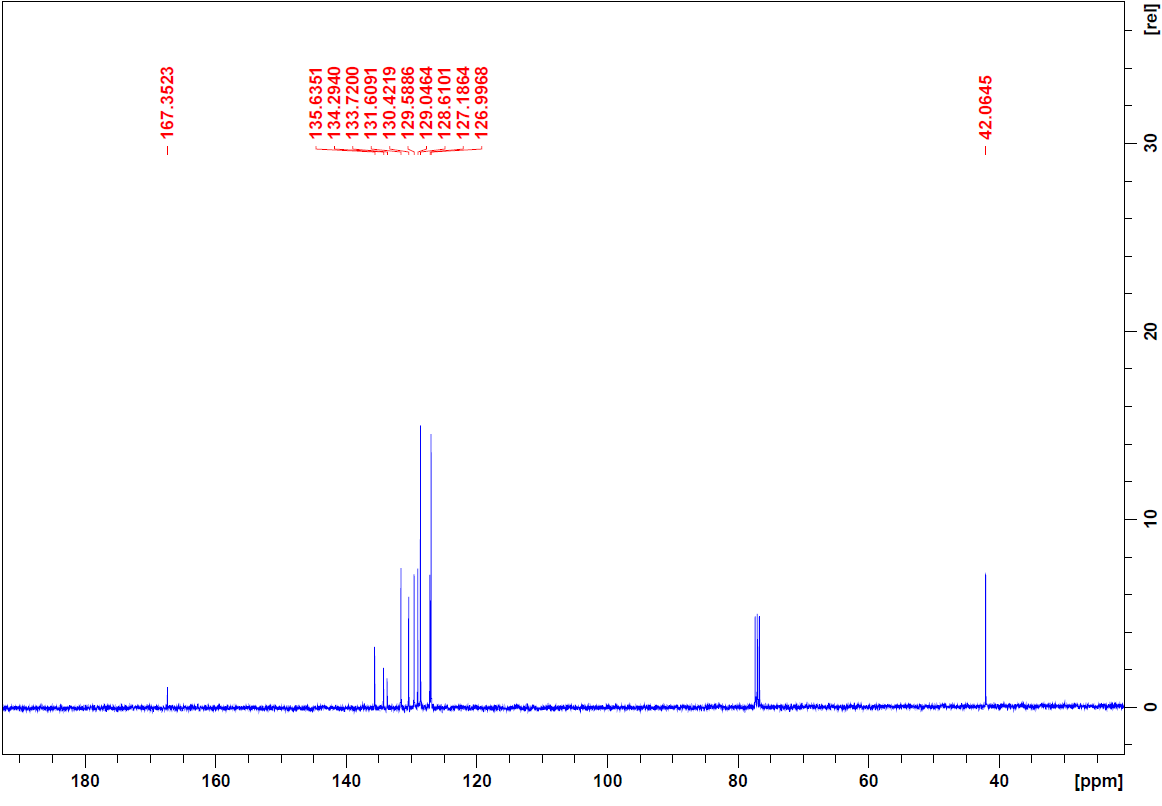


**NMR spectra for 4-chloro-*N*-(phenylmethyl)-benzamide**


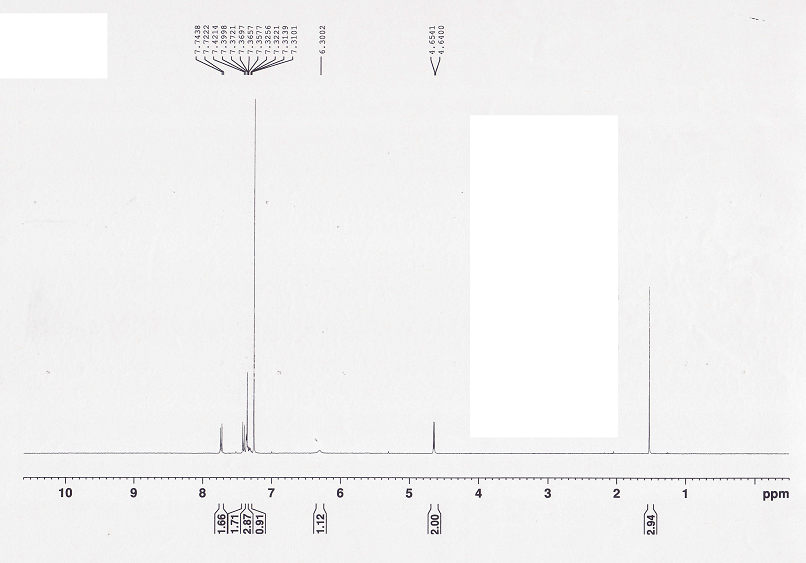


**
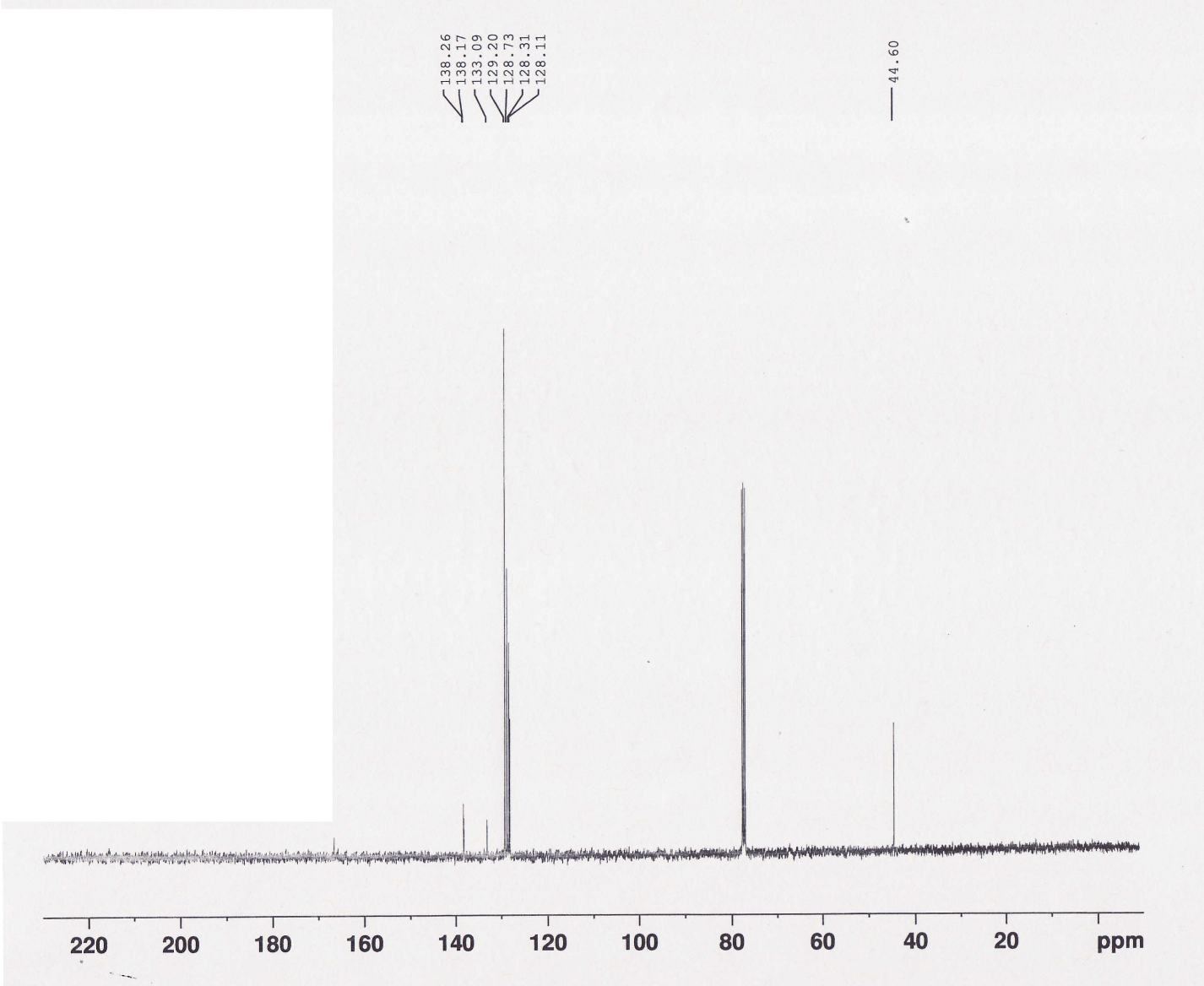
**

**NMR spectra of imides**


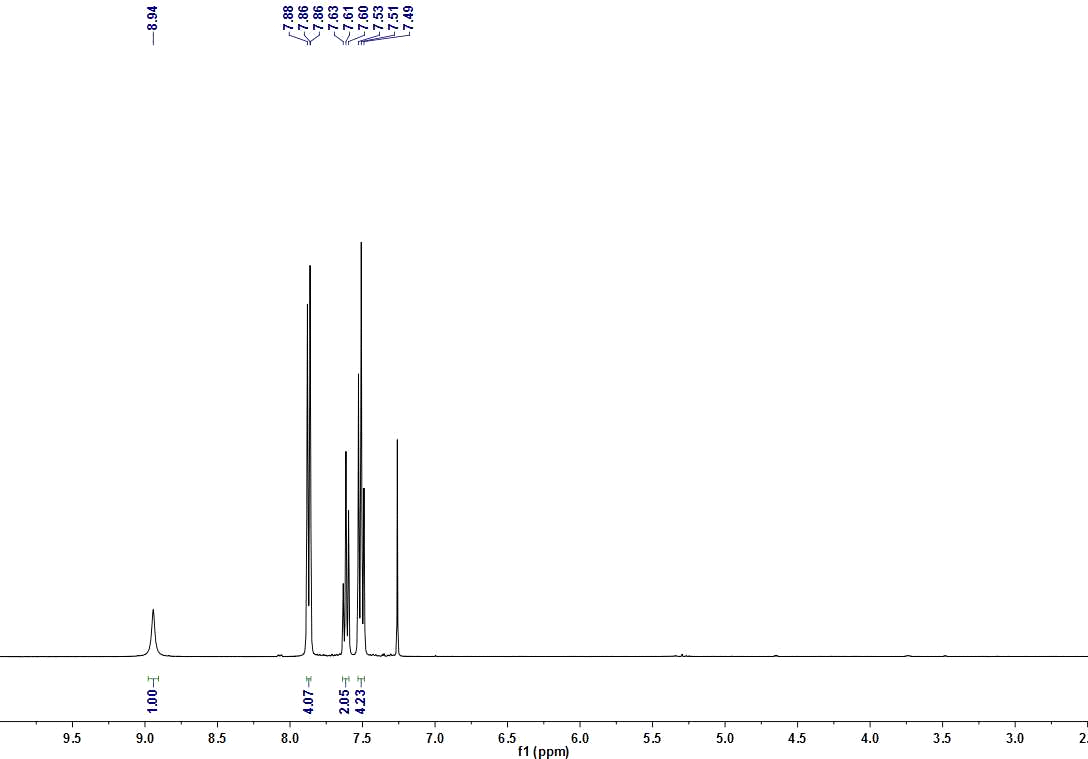

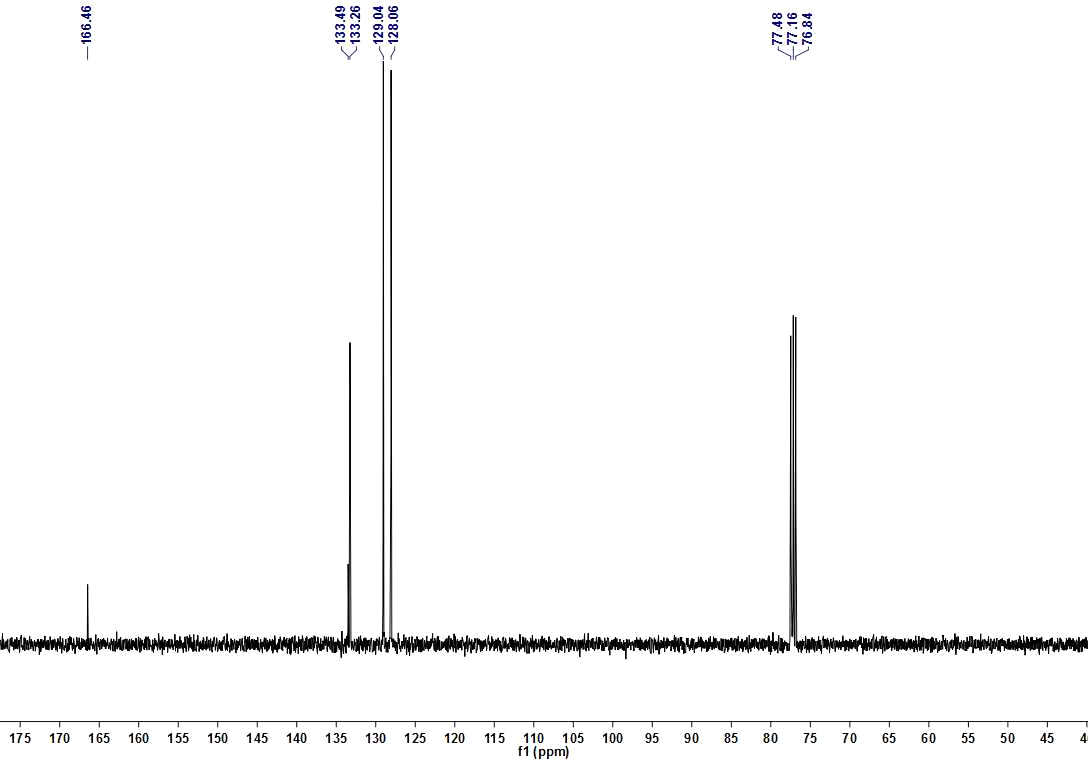

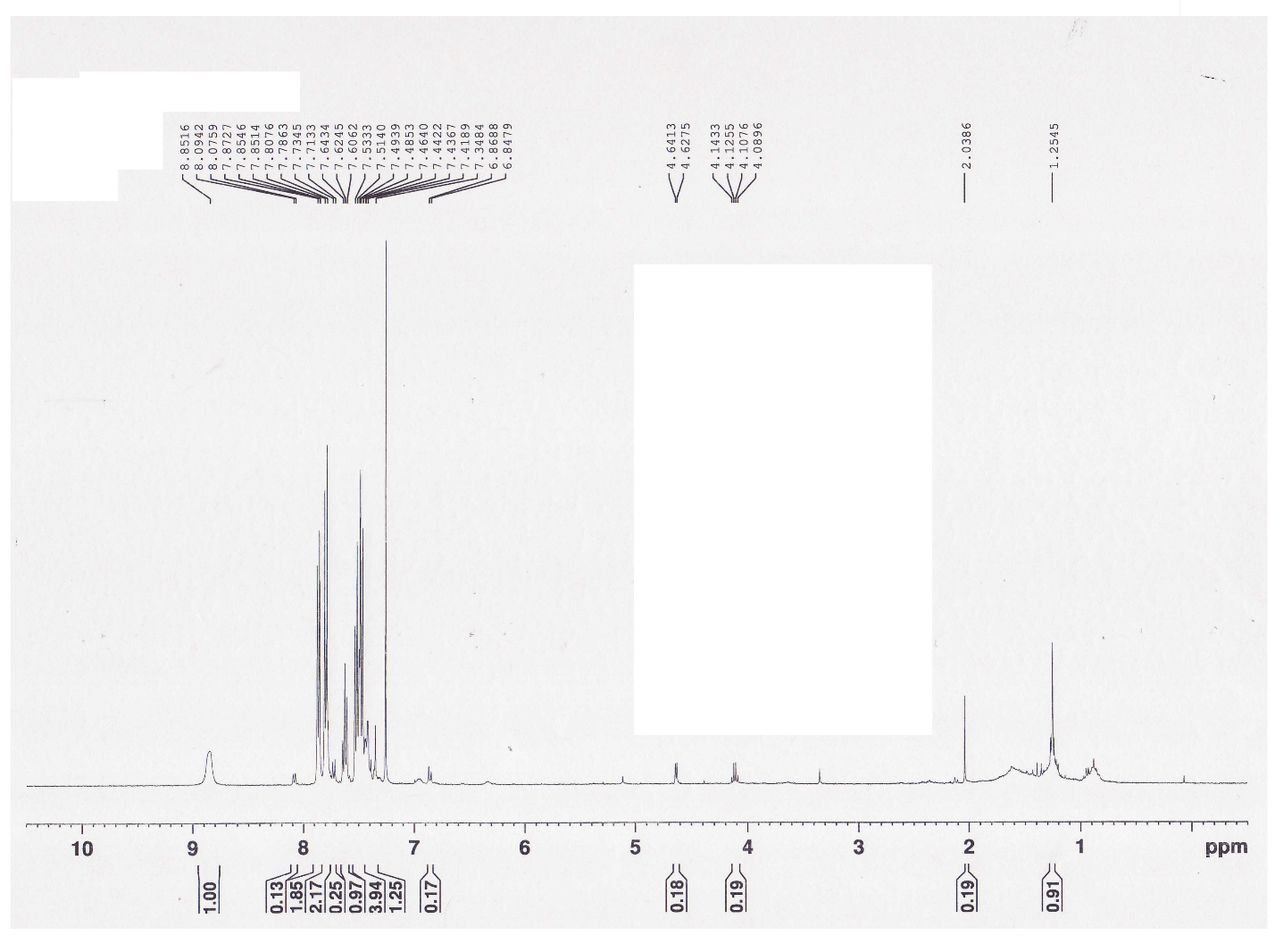


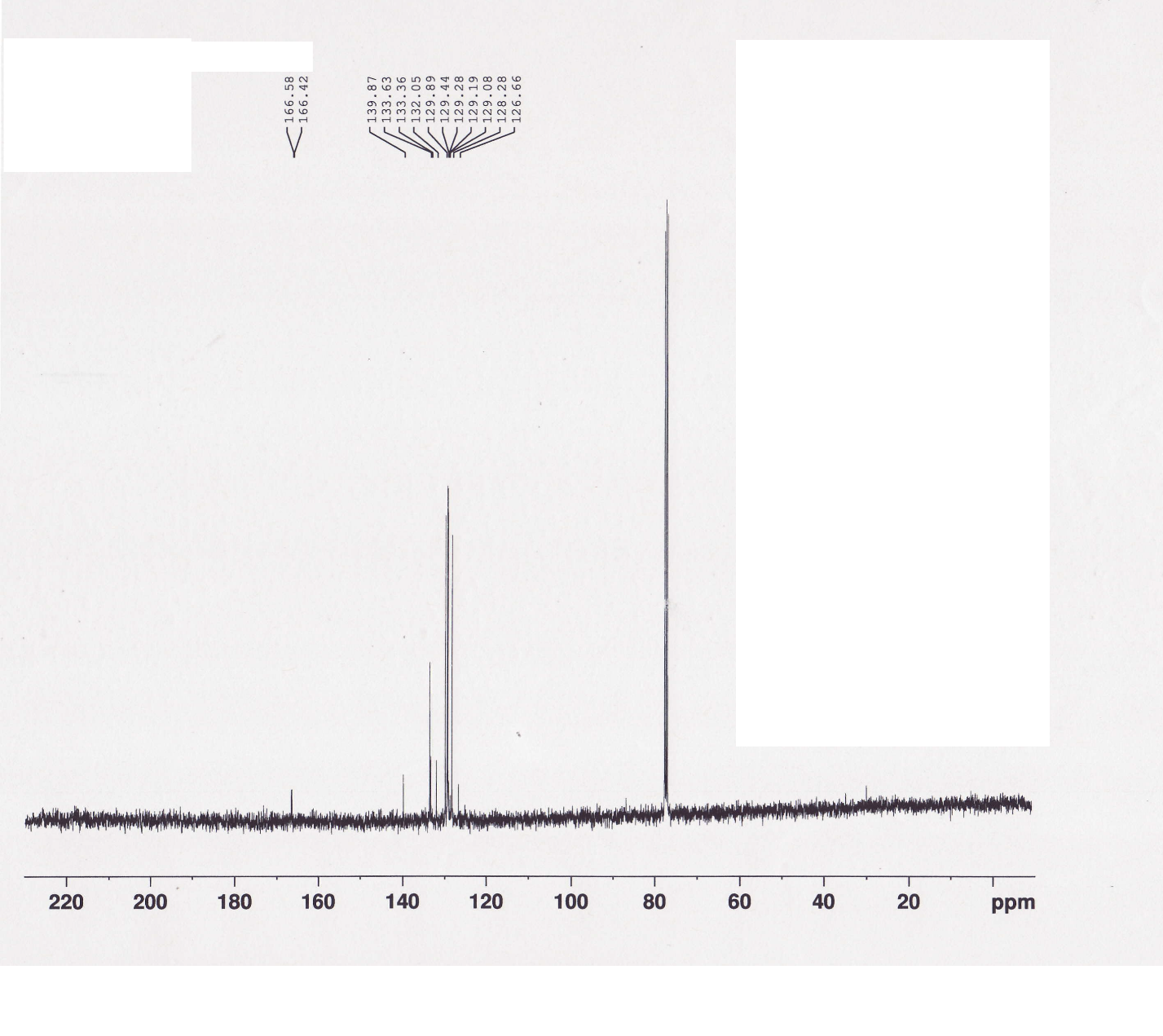


References

1. Burchat, A. F., Chong, J. M. & Nielsen, N. Titration of alkyllithiums with a simple reagent to a blue endpoint. *J. Organomet. Chem.* **542,** 281–283 (1997).

2. Wang, Y., Wu, Z., Li, Q., Zhu, B. & Yu, L. Ruthenium-catalyzed oxidative decyanative cross- coupling of acetonitriles with amines in air: a general access to primary to tertiary amides under mild conditions. *Catal. Sci. Technol* **7,** (2017).

3. Chen, H. *et al.* Fragment-based drug design and identification of HJC0123, a novel orally bioavailable STAT3 inhibitor for cancer therapy. *Eur. J. Med. Chem.* **62,** 498–507 (2013).
